# Hardness Characterization of Simultaneous Aging and Surface Treatment of 3D-Printed Maraging Steel

**DOI:** 10.3390/ma18214830

**Published:** 2025-10-22

**Authors:** Zsuzsa Szabadi Olesnyovicsné, Attila Széll, Richárd Horváth, Mária Berkes Maros, Mihály Réger

**Affiliations:** 1 Doctoral School on Materials Sciences and Technologies, Obuda University, H-1081 Budapest, Hungary; szabadi.zsuzsa@phd.uni-obuda.hu (Z.S.O.); szell.attis@stud.uni-obuda.hu (A.S.); reger.mihaly@uni-obuda.hu (M.R.); 2 Bánki Donát Faculty of Mechanical and Safety Engineering, Obuda University, H-1081 Budapest, Hungary; maros.maria@bgk.uni-obuda.hu

**Keywords:** additive manufacturing, maraging steel, surface treatment, aging, nitriding, PVD coating, surface and in-depth hardness, modified expanding cavity model (ECM)

## Abstract

The primary objective of this research is to simplify and make the industrial manufacturing process of coated maraging steels more economical by combining the advantages of additive manufacturing with simultaneous bulk (aging) and surface (nitriding) treatment in an effective manner. With this aim, preliminary experiments were performed that demonstrated the hardness (and related microstructure) of an as-built MS1 maraging steel, produced by selective laser melting (SLM), is comparable to that of the bulk maraging steel products treated by conventional solution annealing. The direct aging of the solution-annealed and as-built 3D printed maraging steel resulted in similar hardness, indicating that the kinetics of the precipitation hardening process are identical for the steel in both conditions. This assumption was strengthened by a thermodynamic analysis of the kinetics and determination of the activation energy for precipitation hardening using Differential Scanning Calorimetry (DSC) measurements. Industrial target experiments were performed on duplex-coated SLM-printed MS1 steel specimens, which were simultaneously aged and salt-bath nitrided, followed by PVD coating with three different ceramic layers: DLC, CrN, and TiN. For reference, similar duplex-coated samples were used, featuring a bulk Böhler W720 maraging steel substrate that was solution annealed, precipitation hardened, and salt-bath nitrided in separate steps, following conventional procedures. The technological parameters (temperature and time) of the simultaneous nitriding and aging process were optimized by modeling the phase transformations of the entire heat treatment procedure using DSC measurements. A comparison was made based on the in-depth hardness profile estimated by the so-called expanding cavity model (ECM), demonstrating that the hardness of the surface layer of the coated composite material systems is determined solely by the type of the coatings and does not influenced by the type of the applied substrate materials (bulk or 3D printed) or its heat treatment (whether it is a conventional, multi-step treatment or a simultaneous nitriding + aging process). Based on the research work, a proposal is suggested for modernizing and improving the cost-effectiveness of producing aged, duplex-treated, wear-resistant ceramic-coated maraging steel.

## 1. Introduction

Precipitation-hardened maraging steels were developed in the early 1960s [1]. As they possess excellent properties (hardenability, toughness, thermal fatigue resistance, etc.) and are relatively easy to process (simple heat treatment is sufficient, no protective atmosphere is necessary, and they exhibit excellent 3D printability), they can be an attractive choice for molds and dies, provided there are no special requirements. However, maraging steels have some shortcomings in these applications, notably their relatively low wear resistance. According to the literature, the surface coating of maraging steel can increase wear resistance considerably [2]. In this regard, Cajner et al. [3] investigated modification and coating processes with a special focus on increasing wear resistance. Similarly, Hatos et al. [4] showed that the worn area, a quantitative wear indicator, decreased dramatically when maraging steel was nitro-carburized. Therefore, surface treatment (such as nitriding or coating) is an effective way to enhance wear performance.

The first step of the heat treatment process of maraging steel parts produced by conventional methods is solution treatment, to obtain a homogeneous, annealed state (annealing at 780–820 °C, followed by air cooling). The soft, annealed structure consists of iron-nickel martensite with high dislocation density and is substantially free of precipitates. The increase in strength is provided by the precipitates that form during aging at 480–500 °C. The details of the treatments (optimal temperature and time), as well as the expected mechanical properties, can be found in several studies [5,6,7,8,9,10].

A similar heat treatment process is commonly used for maraging steel components produced by additive manufacturing. The effect of heat treatment on the microstructure and mechanical properties of maraging steels produced by selective laser melting was investigated by Bai et al. [11] and Song et al. [12]. Monkova et al. [13] studied the effects of 3D printing direction and heat treatment on the mechanical properties of maraging steel.

Many researchers investigated the use of plasma nitriding to improve the wear and corrosion resistance [14] and the influence of the plasma-nitriding temperature on the microstructure and surface properties [15,16] of 18Ni-300 maraging steel or Inconel 625 steel [17] manufactured by additive manufacturing technology. During SLM, the part is constructed from metal powder melted by a laser, with a particle size of 20 to 40 µm [18,19]. The resulting microstructure is a very fine network of cellular or slightly dendritic needles, formed due to rapid cooling. By modifying the technological parameters, structures with different anisotropies can be produced, and compactness approaching 100% (porosity approaching 0%) can be achieved. Koutny et al. [20] investigated 3D-printed maraging steel with two SLM machines and achieved 99.9% relative density. Similarly, Mugwagwa et al. [21] reached relative densities of 99.6% and 99.4%. Demir and Previtali [22] also demonstrated that with optimized process parameters, steel with porosity below 1% can be produced.

The microstructure and mechanical properties of the reference (i.e., the solution-treated) and 3D-printed maraging steels show several similarities [18,23,24,25]. The conventional solution treatment results in a homogeneous solid solution with a martensitic structure. Similarly, rapid cooling after laser melting also produces a solid solution. The primary differences between solid solutions produced using these two methods are grain size and shape, dislocation density, and residual stress levels. Additionally, partial aging can occur in 3D printed parts due to subsequent heating cycles caused by laser beam scans, resulting in the formation of small amounts of austenite (1–3%) within the matrix [26].

Many researchers have investigated the simultaneous aging and surface treatment (nitriding, carburizing) of steel, as it offers significant advantages from an industrial perspective (e.g., time, cost, and energy reduction). In these studies, most researchers employed gas nitriding on stainless steel [27] or maraging steel [28], and similarly, plasma nitriding was used on plastic mold steel [29], stainless steel [30], or maraging steel [31]. In recent years, the simultaneous aging and surface treatment of additively manufactured 18Ni300 maraging steel (3D printed) has also been investigated [32]. The concept of simultaneous nitriding and aging is derived from the practical observation that the temperature range of industrially applied salt bath nitriding (480–630 °C) [33] coincides with the temperature range for steel aging (480–530 °C). Therefore, by coordinating the temperatures and durations required for aging and nitriding, the two treatments can be carried out simultaneously. The surface treatment applied in this study consists of two steps, nitriding and physical vapor deposition (PVD) coating, called duplex treatment, as nitriding ensures a transition layer for better connection and load distribution between the softer core material and the more rigid PVD coating efficiently improving the wear resistance [34,35,36,37]. As deposition of the investigated PVD layers (DLC, CrN, and TiN) requires a far lower temperature, this final treatment does not affect the results of aging or nitriding.

The current research aims at answering the question of whether the technological process of a duplex-coated (nitride + PVD coated) maraging steel product having a conventionally heat treated—solution-annealed and precipitation hardened—martensitic structure, produced from a bulk matrix material, is equivalent to that, when replacing the raw material with 3D printed (SLM) maraging steel and the industrial heat treatment process is simplified by using simultaneous nitriding and precipitation hardening before the PVD coating instead of the much more time-consuming and costly standard multi-step heat treatment process.

To answer these questions, a complete industrial heat treatment process for producing duplex-coated samples with a maraging steel substrate with two different initial conditions was performed. To design the optimal technological parameters for this target test, several preliminary tests were conducted. Among others, the effect of bulk heat treatment on the structure and hardness of the 3D-printed maraging steel samples was investigated. Using DSC tests, the activation energy of the precipitation hardening process was determined, the process kinetics were analyzed, the optimal duration of the planned simultaneous nitriding and aging process was identified, and the evolution of hardness during aging was examined. Additionally, the complete industrial heat treatment process was modeled experimentally by DSC measurements. Beyond this DSC-based optimization, recent studies suggest that data-driven approaches can further accelerate parameter selection and improve reliability in industrial heat treatment [38].

The study focuses first on the direct aging of 3D printed maraging steels in their as-built state, without prior solution treatment. Then, the technological criteria of simultaneous aging and nitriding are discussed. Finally, we present the experiences and results of bulk heat treatment, nitriding, and PVD coating of 3D printed samples implemented under industrial conditions. Because of the surface topography and near-surface structure of the samples, special attention was paid to the precise determination of the in-depth hardness distribution. A special procedure was developed for the estimation of in-depth hardness distribution, both for nitride and duplex coatings, by applying the modified expanding cavity method.

## 2. Materials and Methods

### 2.1. Specimens

During the different tests, 3D-printed and conventional bulk specimens were used. The printed samples were produced by SLM using an EOS M 290 3D printer (EOS GmbH, Robert-Stirling-Ring 1, 82152 Krailing, Germany) with the following printing parameters. Laser power: 300 W; scanning speed: 750 mm/s; scanning interval: 0.12 mm; powder thickness: 55 μm. The metal powder grade used in additive manufacturing was EOS MS1 maraging steel (Table 1). Based on its chemical composition, it has the same quality as 18Ni (300). Based on metallographic examination of the samples, we estimated porosity to be lower than 0.5%. For heat treatment experiments, 4 mm thick, disk-shaped pieces were machined from a 3D-printed standard (∅25 × 100 mm) Jominy-shaped bar.

In the experimental work, specimens of the reference material (Böhler W720, Böhler GmbH., Korntal-Münchingen, Germany, solution annealed, Table 1) were used for comparison. They had a similar thickness to the 3D printed ones, cut by sawing from a *D* = 30 mm diameter, as-received commercially available hot-rolled steel rods. The opposite flat surfaces were simultaneously ground to obtain parallel surfaces for the different measurements.

For DSC tests, samples with a diameter of 4.9 mm and a height of 1.5 mm were prepared from both 3D-printed EOS MS1 and bulk BöhlerW720 base materials.

The study involved a series of pre-tests and target tests using different specimens, listed and identified according to the experimental matrix (Appendix A).

### 2.2. Technologies, Equipment, Parameters of Heat Treatments, and Testing

Within the framework of industrial cooperation, we conducted simultaneous heat treatment experiments to produce nitrided and PVD-coated samples. As-built 3D printed maraging steel specimens produced by SLM were aged, nitrided, and PVD coated in the following steps: preheating to 380 °C for 60 min, nitriding at 530 °C for 25 min, and PVD coating at 300 °C for 6–8 h. The expected thickness of the nitrided layer was 10–15 µm. Three different PVD coatings of a thickness of 3–6 µm were deposited onto the nitrided surface of the samples. The types of PVD coatings were selected according to typical tool steel applications as follows:

- DLC: diamond-like hydrogenated amorphous carbon with a tungsten carbide base layer, with an expected hardness of 2500–4000 HV;
- CrN: chromium nitride base layer, with an expected hardness of 1500–3000 HV;
- TiN: titanium nitride base layer, with an expected hardness of 2000–3500 HV.

For surface characterization of the aged and coated samples, surface hardness, in-depth hardness distribution, and surface roughness were investigated.

Surface hardness is of paramount importance because it strongly influences the wear performance of the coated material system. In the case of a coated sample, the surface hardness strongly depends on the applied load, due to the so-called substrate effect. In the cases investigated, the matrix is significantly softer than the coating; therefore, the higher the load, the lower the measured surface hardness, referred to as composite hardness, which reflects the joint response to the applied load of the composite material system consisting of the coating and substrate together. Sets of surface hardness tests with increasing loads were performed. The surface hardness of the samples was measured with the Vickers method, for microhardness and low-load hardness tests, loads of 0.05, 0.1, 0.2, 0.5, 1, and 2 kgf, and for macrohardness tests, loads of 5, 10, 20, 30, 40, 60, 100, and 120 kgf were used in the case of nitrided and duplex- (nitrided and PVD) coated samples. Hardness was tested with a Wilson VH1102 (Buehler microhardness tester, 41 Waukegan Road, Lake Bluff, IL, USA), a Zwick 3212 (ZwickRoell GmbH & Co. KG micro- and macrohardness tester, Ulm, Germany), and a conventional macrohardness tester (WPM HPO 250, WMW, Leipzig, Germany).

The surface roughness (*R_a_* and *R_z_* parameters [39]) of the samples was determined both in the nitrided and the duplex-treated (nitrided plus PVD-coated) conditions by the Mitutoyo SJ310 (Mitutoyo Corporation, Tokyo, Japan) device.

The thickness and topography of the coatings were examined by optical and scanning electron microscopy. The applied equipment was an OLYMPUS DSX1000 opto-digital microscope (Olympus Europa SE & Co. KG, Hamburg, Germany) and an electron microscope (JSM 5310, Jeol Ltd., Tokyo, Japan). In addition, coating thickness was also determined by the ball-cratering method (Calotest) on the as-coated surface of the specimens using a Calotester (CSM Calotest Compact, Anton Paar GmbH, Graz, Austria). The cross-section samples for microscopic analyses were ground, polished, and etched by 2% Nital before the examination.

DSC measurements were accomplished using a PerkinElmer DSC 8000 (PerkinElmer, Inc., 940 Winter Street, Waltham, MA, USA) device.

### 2.3. Characterization of the Surface Hardness of the Nitrided and Coated Samples Using the Modified ECM

The surface hardness, as mentioned before, can be regarded as a composite hardness, which can be interpreted and calculated as a volumetric characteristic feature. According to Burnett’s model, Equation (1) creates a physical connection between the half-diagonal of the indentation (*a*) and the size (*b*) of the plastic zone developing under the indentation [40,41].

(1) $$b=a{\left(\frac{E}{H_{s}}\right)}^{\frac{1}{p}}\times c{\mathrm{ot}}^{\frac{1}{3}}\varphi$$

where *E* is the elastic modulus (GPa), *H_s_* is surface hardness (GPa), *p* is a constant between 2 and 3 (in this study, *p* = 3), and *φ* is the indenter semi-angle (148°/2 = 74°). In the case of a given size of the plastic zone below the Vickers indentation, if the subsurface hardness distribution, i.e., hardness values belonging to the different *i*-th spherical segment of plastic zone are known, the surface composite hardness *H_comp_* obtained at different normal loads, i.e., *H_s_*(*h*) to varying depths of indentation of a PVD-coated duplex-treated tool steel, can be calculated by Equation (2). This relationship is an improved version of Burnett’s model, as suggested by Ichimura et al. [42,43].

(2) $$H_{comp}=H_{s}\left(h\right)=\left(\frac{V_{f}}{V_{0}}\right)H_{f}+\sum_{i=1}^{n}\left(\frac{V_{i}}{V_{0}}\right)H_{i},$$

where *V_f_* and *H_f_* are the volume and hardness of the surface coating, respectively; *V_i_* and *H_i_* are the volume and characteristic hardness of the *i*-th spherical segment below the surface indentation, respectively; and *V*_0_ is the volume of the plastic zone of indentation with depth of *h* created by the given load. The left-hand side term in the equation refers to the coating, while the right-hand side term accounts for the hardness of the nitrided layer.

The question is, how is the in-depth hardness distribution defined? It can be measured directly at predefined depth steps below the surface or approximated based on theoretical considerations. We followed the second procedure.

The load dependency of the composite hardness, HV = HV(*F*), characterizing the entire coated system, is well understood. The surface hardness versus load function and the in-depth hardness versus depth function are related in a complex manner. They can be converted into each other using the so-called modified expanding cavity model (ECM), as described in a previous work [44], which provides a detailed discussion of the theoretical background and calculation method. In this study, a simplified method based on the ECM was employed to estimate the in-depth hardness distribution functions in the subsurface region of the coated material systems. According to the modified ECM, the in-depth hardness distribution, *H_d_*(*x*), in a nitrided layer without coating can be approximated by a sigmoidal function as defined by Equation (3) [44].

(3) $$H_{d}\left(x\right)=A_{1}+\frac{A_{1}-A_{2}}{1+e^{\frac{x-x_{0}}{dx}}}$$

where *A*_1_ is the upper and *A*_2_ is the lower bound of the in-depth hardness function, *x*_0_ is the *x* coordinate of the point of inflection, and *dx* is the so-called “time constant” describing the stretching of the function along the *x*-axis.

The parameters of Equation (3) have a physical meaning. Therefore, the range of studied values can be specified for a given material and surface treatment. Parameter *A*_1_ represents the maximum value of the in-depth hardness distribution, and its expected range can be estimated for commonly used surface treatments. The hardness of the matrix (substrate core hardness), denoted as *A*_2_, is easily defined and can be treated as a constant. The free variable *x*_0_ indicates the distance of the inflection point of the curve from the surface, and its value falls within the expected layer thickness range. The constant *dx* can be calculated from the *x*_0_*/dx* ratio, which typically ranges from 2 to 40 for surface layers. A higher *x*_0_*/dx* ratio indicates a steeper change in hardness near the inflection point.

In the current study, the surface hardness as a function of the applied load was assessed using an in-depth hardness distribution function derived from the ECM, whose parameters were estimated from measured local in-depth hardness values.

**Figure 1:**
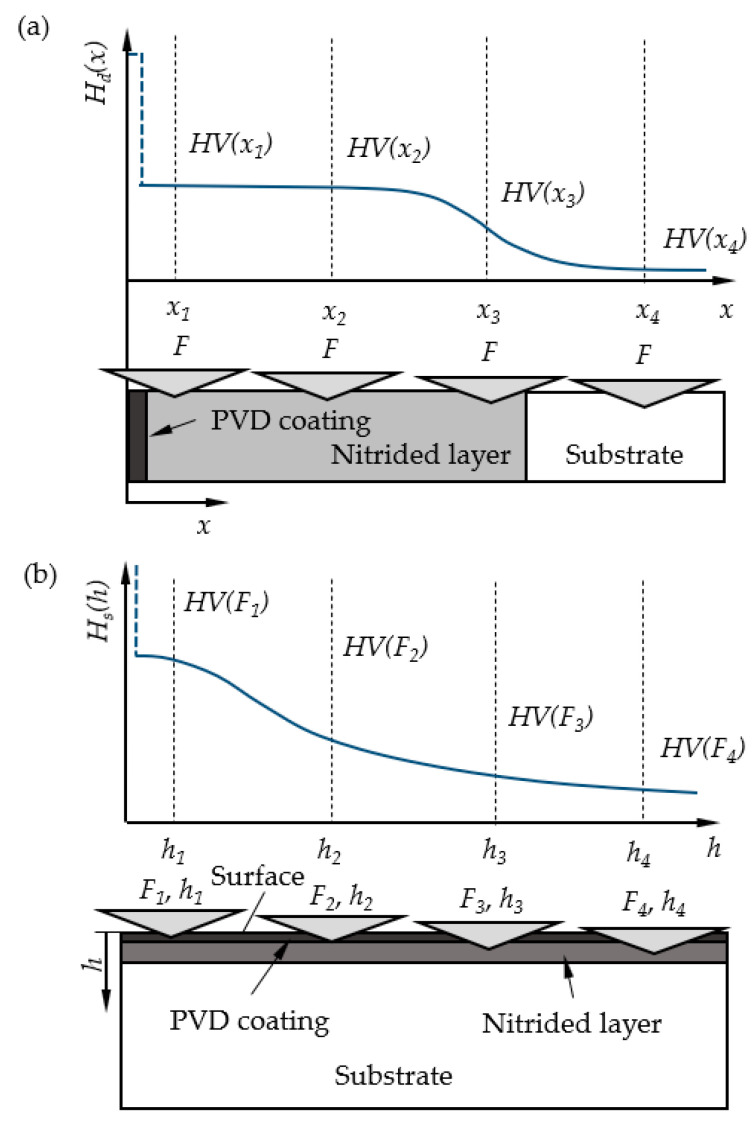
Sketch of the measurement set-up and the hardness distribution of duplex coated (nitrided + PVD coated) samples assessed by the ECM in case of in-depth hardness (**a**), and surface hardness (**b**) measurements. The hardness distribution in the (**a**) part of the figure is given as a function of distance from the surface, while in the (**b**) part, it is a function of the indentation depth or indentation load. The coating constant hardness region is indicated by a dotted line.

For characterizing the duplex coated samples a bimodal (stepwise) hardness function was used, consisting of, on the one hand, a thinner (3–5 µm), constant hardness region corresponding to the PVD coating, and, on the other hand, a region of gradually decreasing in-depth hardness representing the thicker nitrided subsurface layer (Figure 1).

The in-depth hardness distribution function *H_d_*(*x*) characterizing the nitrided layer is approximated by the ECM (Equation (3) based on local *HV*_1_, *HV*_2_, *HV*_3_, *HV*_4_…*HV_i_* hardness values measured by constant load at different *x_i_* depths in the subsurface region (Figure 1a)). Then, the coating surface hardness is calculated using Equations (1) and (2) as a function of the applied load. The surface hardness *H_s_*(*h*) and the in-depth hardness *H_d_*(*x*) functions are related, as the hardness of the same surface layer is investigated with hardness tests in different directions and positions.

The physical interrelation of the *H_d_*(*x*) and *H_s_*(*h*) provides an inverse procedure for the calculations, as well. In the case of measuring the surface hardness with different normal loads, the value of the surface hardness function, *H_s_*(*h*), decreases with an increasing load (*F_i_*), i.e., with increasing indentation depth, *h_i_* (Figure 1b). In this case, the input data for the calculations are the *HV*(*F_i_*) values, and the in-depth hardness distribution, represented by the *H_d_*(*x*) function, is assessed.

In the following sections, the consecutive steps and results of the experimental work are presented, divided into three main parts: pre-tests, target test design, and implementation of the industrially realized target tests. The experimental matrix, which includes details of all pre-tests and target tests, is also found in Appendix A.

## 3. Experimental Work

### 3.1. Pre-Tests to Investigate the Effect of the Direct Aging of Parts Produced by SLM

We performed preliminary experiments to compare the effect of aging by measuring the hardness of 3D printed samples in both as-built and solution-treated (annealed) conditions (Table 2). The data in Table 2 indicate that the initial hardness of the maraging 3D printed steel in the untreated, as-built (PP1) condition was greater than after solution annealing (PP4). However, after aging, the measured hardness was closely identical for the two series of samples, as seen from the data of PP2 vs. PP5, or PP3 vs. PP6, independently of their initial condition, whether they were as-built or solution-annealed. In addition, increasing the aging time by almost two times, a similar (6%) increase in hardness in both series of samples (PP2 vs. PP3, and PP4 vs. PP6) was observed. The standard deviation of the hardness of the untreated specimens was higher than that of the solution-annealed specimens.

We performed DSC (I) tests [45] to compare the kinetics of the hardening process of the as-built and the solution-annealed 3D printed maraging steel, using continuous heating experiments up to a temperature of 600 °C, with heating rates of 2, 5, and 10 K/min, and the temperature range and activation energy of the aging process were determined.

In the temperature range used for aging maraging steels, two exothermic peaks (Peak 1 and Peak 2) and two endothermic peaks are generally identified. According to the literature [46,47,48,49], the first heat effect, occurring between 350 and 450 °C (Peak 1), can be related partly to the recovery of martensite and partly to the formation of the new, coherent intermetallic phases. The second exothermic reaction (Peak 2) appearing between 470 and 530 °C can be interpreted as a result of the coarsening of the intermetallic particles, accompanied by an increase in their incoherency, and the partial conversion of martensite into austenite. The endothermic peaks identified at higher temperatures correspond to the transformation of martensite into austenite, the dissolution of precipitates, and recrystallization reactions [49]. The activation energies (Table 3) were calculated using Kissinger’s method [50] for different heating rates.

The temperature ranges of the exothermic peaks for the as-built and the solution-annealed samples are very similar. The different activation energies of Peak 1 for samples of different conditions indicate some dissimilarity in the kinetics of the precipitation processes.

We also performed DSC tests with different interrupted heating to determine the relationship between the thermal history and the change in hardness. This measurement technique is based on the assumption that the diffusion processes occurring during the slow heating or isothermal holding period characteristic of DSC testing are not significantly influenced by the short-term rapid cooling and reheating periods that arise between them. Accordingly, if the heat treatment of a sample is interrupted at a given moment by rapid cooling, hardness at room temperature can be determined. Then the heat treatment can be continued with the fast reheating of the same sample.

In the first set of the interrupted heating experiments, represented by DSC (II) tests, the sample was heated to 600 °C at a heating rate of 10 K/min, and heating was interrupted at 450, 500, and 550 °C. In the following trial, the DSC (III) test series was conducted with heating interrupted at 50 °C increments between 150 and 600 °C. The subsequent DSC (IV) tests, involving heating three samples individually to 500, 550, and 600 °C, followed by rapid cooling, were performed to verify the results of the DSC (II) test series. The hardness of the heated samples was determined in each case by HV5 hardness testing.

**Figure 2:**
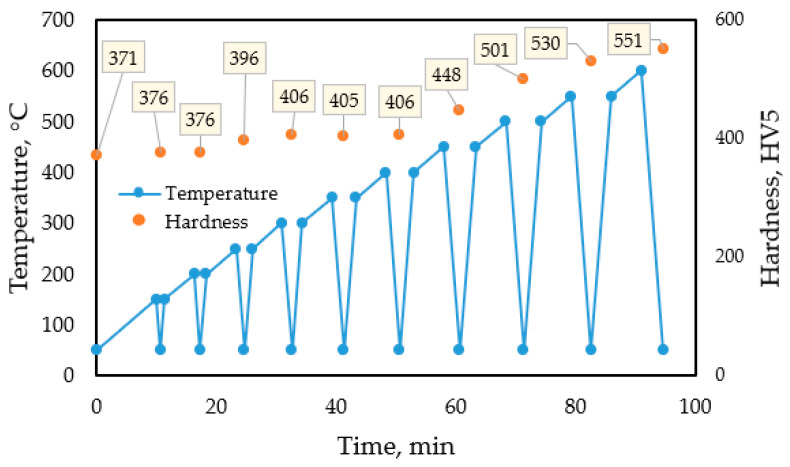
Time-temperature diagram of the interrupted DSC (III) experiments, presenting the hardness values measured after rapidly cooling the samples to room temperature at each 50 °C temperature increment step.

**Figure 3:**
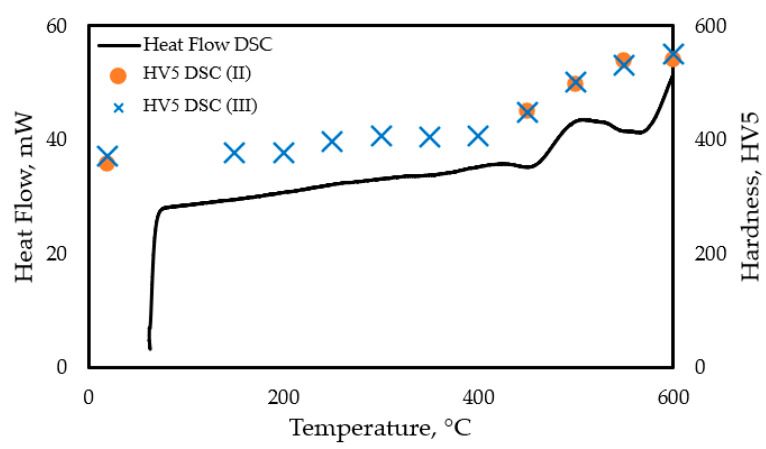
Heat flow of the DSC (IV) (continuous heating) and hardness values measured in the interrupted DSC (II) and DSC (III) experiments.

Figure 2 shows the time–temperature diagram for the DSC (III), i.e., the frequently interrupted DSC test series, indicating the HV5 values measured in each step of the interrupted heat treatment process. Figure 3 illustrates the heat flux and hardness values obtained from the interrupted test for the as-built samples.

Based on the experiences gained from the pre-tests, it can be established that the optimal aging temperature providing a maximum hardness is around *T* = 480–550 °C. 3D-printed maraging steel samples in their as-built condition can be used for the target tests instead of the common volume-treated bulk maraging steel specimens.

### 3.2. Designing the Target Test for the Simultaneous Heat Treatment of Parts Produced by SLM

Simultaneous aging and salt-bath nitriding experiments were planned to be conducted on the as-built 3D-printed maraging steel specimens under industrial conditions. According to these conditions, the specimens are soaked in the salt bath at temperatures between 350 and 400 °C before being nitrided in a cyanide-free salt bath at temperatures ranging from 480 to 630 °C. In practice, a bath temperature of 580 °C is typically used for nitriding, which exceeds the optimal temperature of 480 °C for aging. Thus, the temperature of the simultaneous nitriding and aging treatment had to be defined as a compromise between the optimal nitriding temperature and the optimal aging temperature. Accordingly, it was chosen to be 530 °C. Temperatures lower than this could cause difficulties (undesirable increase in the salt-bath viscosity, reduced efficiency of diffusion, etc.). Nevertheless, this temperature is still higher than the optimum one for the precipitation hardening process.

**Figure 4:**
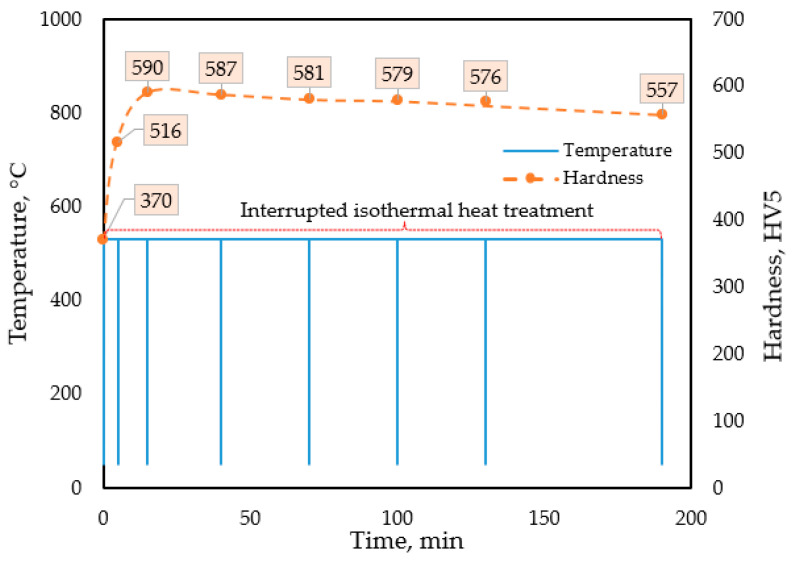
Time-temperature diagram for the DSC (V) test involving quasi-isothermal soaking at 530 °C of the as-built SLM-produced maraging steel, and hardness values measured at each interruption step after rapid cooling to room temperature.

Therefore, we studied the kinetics of the aging process at a temperature of 530 °C by performing the quasi-isothermal DSC (V) tests, which were interrupted at predefined time intervals. Within the temperature interval represented by the soaking and room temperatures, the heating and cooling rates were 150 K/s, which were high enough to avoid undesired diffusional processes during heating and cooling. The aging process was repeatedly interrupted at total soaking times of *t_soak_* = 5, 10, 15, 30, 60, 90, 120, 180 min. Figure 4 shows the time-temperature diagram of this set of experiments, along with the hardness measured at room temperature during the interruption periods.

For an optimal precipitation-hardened structure, the desired duration of the salt-bath nitriding at 530 °C falls between 15 and 25 min. In this case, the thickness of the nitrided layer is expected to be thin, 10–15 µm. For a thicker nitrided layer, longer soaking times are necessary. However, in this case, some overaging of the precipitates in the matrix material will occur, which may decrease the hardness by around 30 HV.

**Figure 5:**
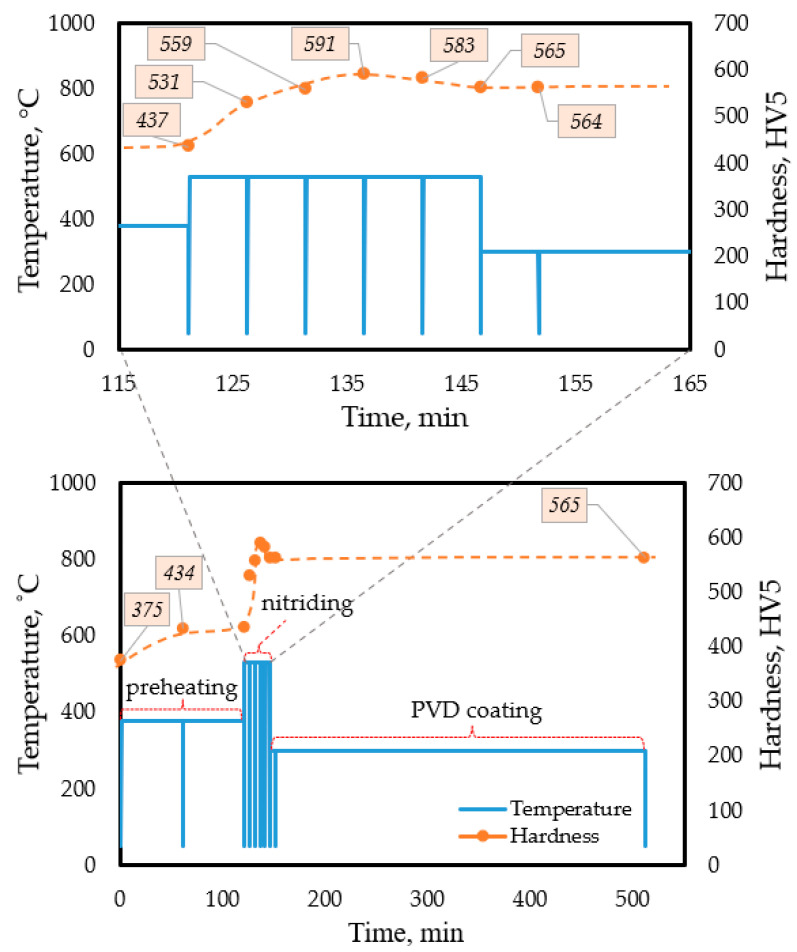
Time-temperature diagram of the DSC (VI) test, modeling the planned complete heat treatment cycle, including preheating, nitriding, and PVD coating, and assigning the measured hardness values at different stages.

To design an appropriate simultaneous (nitriding + aging) heat treatment, we performed DSC (VI) tests, modeling the entire industrial heat treatment cycle accomplished on as-printed samples. The technology consists of three main steps, as described before. The first step is to preheat the samples for a maximum of 2 h at a temperature of up to 380 °C. This step is followed by nitriding at 530 °C for a maximum of 25 min, allowing the aging process to co-occur. As a final step of the treatment, PVD coating is performed for a maximum of 8 h at a maximum of 300 °C. To monitor the hardness evolution over the entire cycle, we interrupted the modeling heat treatment process at certain times. The interruption was implemented at shorter time intervals during nitriding to track the significant change in hardness, a characteristic of this stage (Figure 5).

The maximum hardness value during simultaneous aging and nitriding treatment, achieved with the technological parameters of the DSC (VI) test, is 590 HV, reached within 15 min from the start of the nitriding step. A slight decrease happens in the remaining 10 min while holding the sample at 530 °C. The thermal effect to which the samples are exposed during the subsequent long-lasting PVD coating process at 300 °C does not significantly affect the final hardness of the substrate material.

### 3.3. The Industrial Complex Heat Treatment Target Test Procedure

#### 3.3.1. Conditions of the Performed Industrial Treatment

The objective of the target test in industrial circumstances was to compare the surface layer architecture and hardness distribution in duplex-coated samples with three different ceramic coatings (DLC, CrN, and TiN) deposited on an as-built, SLM-printed MS1 maraging steel and a bulk, solution-annealed W720 maraging steel substrate.

The steps and planned condition of this experiment were, as follows: preheating at a temperature of *T* = 380 °C, with a holding time of *t* = 2 h, then simultaneous nitriding and aging (precipitation hardening) at a temperature of *T* = 530 °C, with a holding time of *t* = 25 min, followed by PVD process producing the three different coatings at a temperature of *T* = 300 °C for holding time of *t* = 8 h.

It should be noted that, while the planned holding time for preheating at 380 °C was 2 h (as shown in Figure 5), in the actual industrial experiments, due to practical reasons, we were able to accomplish only 1 h of holding (a more typical duration in industrial practice). Due to this circumstance, the expected core hardness of the substrates may be slightly lower than shown in Figure 5.

#### 3.3.2. Characterizing the Architecture of the Duplex-Coated Samples

**Figure 6:**
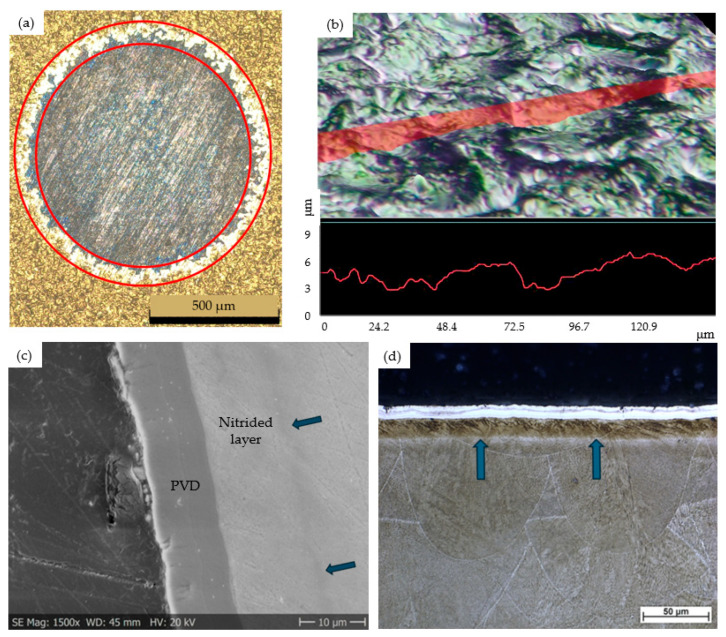
Microstructural and surface characterization of PVD coated samples: (**a**) TiN layer thickness determination by OM on the image of the worn crater produced by Calotest on the PT3 sample; (**b**) surface characterization of DLC coating by 3D OM on PT1 sample; (**c**) surface area of BT2 sample (scanning electronmicroscope); (**d**) surface area of BT2 sample (optical microscope).

The surface layer of the duplex-coated specimens was characterized by the thickness of the nitrided layer and coatings, as determined by optical and scanning electron microscopy, as well as by the roughness parameters R_a_ and R_z_. Coating thickness measurements on the DLC, CrN, and TiN PVD coatings were also performed using Calotest. This method produced a worn crater that allowed for the clear distinction of different layer sections by optical and scanning electron microscopic examination (Figure 6a,b).

The surface roughness of the nitrided samples was significantly degraded by roughly an order of magnitude due to the PVD process. The surface roughness and topography of samples labeled PT and BT were similar, with *R_z_* values ranging from 3 to 4 μm. For example, Figure 6c shows the 3D OM image of the surface of sample PT1, a duplex DLC coating on a 3D printed substrate. A plane perpendicular to the surface is marked in red on the upper part of the image in Figure 6c. The line of intersection of the plane and the coating, i.e., the corresponding surface profile, is illustrated in the bottom section of the image.

In addition, the substrate matrix material was characterized by the core hardness (HV10) values. These characteristics of the samples for the as-printed (PT) and the solution annealed bulk (BT) groups are summarized in Table 4.

The surface layer characteristics indicate that there is no significant difference between the nitrided layer and coating formation processes of the P and B series of samples under industrial conditions. The nitrided layer thickness values, measured by SEM, fell within the range of 9–11 µm and were identical for both the printed and bulk samples. The DLC and TiN PVD layer thickness falls into the range of 1.9–2.6 µm, while that of the CrN coating is 7.1–7.8 µm (Figure 6b). The layer thickness and surface hardness of the as-built 3D printed and bulk, solution-treated specimens were very similar, regardless of whether the matrix material is 3D printed or a traditional bulk maraging steel.

The average core hardness of the samples after industrial preheating, nitriding, and PVD treatment was closely identical for each sample, regardless of the initial substrate condition. The whole set of target test samples can be characterized by an average core hardness of 535 HV5 (Table 4) measured in the middle of the sample.

#### 3.3.3. Determining the Case Depth of the Nitrided Layer of the Target Samples by Estimating the In-Depth Hardness Distribution

The thickness of the nitrided layer can be interpreted in different ways, and methods employed for determining the depth of the case can be either chemical, mechanical, or visual [51]. For example, the nitrided layer depth can be identified as the thickness of the diffusion zone, *x_v_*, which is a visible boundary line in the optical or scanning electron microscopy image of the nitrided cross-section.

**Figure 7:**
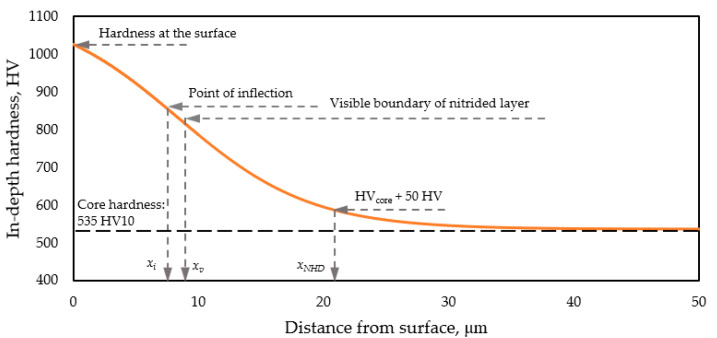
The in-depth hardness function defined for the nitrided layer, and the determination of nitrided layer thicknesses according to different practices.

According to the most common practices, determination is based on the characteristic points of the in-depth hardness distribution curve, also known as the subsurface hardness profile (Figure 7). Based on this curve, the nitrided layer thickness is most simply characterized by the depth of the hardness profile’s inflection point, *x_i_*. The feature referred to as nitriding hardness depth, denoted by NHD according to standard [51], is defined as the depth at which the in-depth hardness reaches a value equal to the matrix core hardness plus 50 HV, and will be here denoted as *x_NHD_*. The investigated metrics (inflection point, NHD) are also consistent with laboratory practices in other fields. For example, in the case of railway materials [52], the extent of the heat-affected zone (HAZ) can be quantified using microhardness curves, similar to the characterization of nitrided surfaces [53].

In the following, we present the procedure used to estimate the hardness distribution developed in the very thin nitrided layer, represented by the red curve in Figure 7. According to Table 4, the thickness of the nitrided layer, as determined by OM and SEM, is between 9 and 11 µm. The in-depth hardness distribution of such a thin layer cannot be directly determined on a metallographic cross-section (perpendicular to the surface) using microhardness measurements, since the size of the microhardness indentations is comparable to the thickness of the nitrided layer (e.g., at 750 HV with a load of 0.02 kgf = 0.196 N, the diagonal of the Vickers indentation is approximately 7 μm).

**Figure 8:**
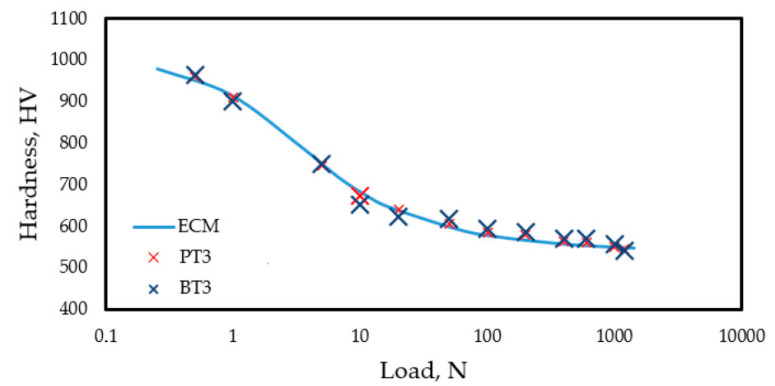
Surface hardness function in the nitrided layer of the 3D-printed (PT3) and bulk (BT3).

Therefore, the in-depth hardness distribution was estimated by measuring the surface hardness of the nitrided samples under various loads. Surface hardness measurements were carried out using normal loads ranging from 0.5 to 1000 N (see Figure 8), prior to PVD coating, on nitrided samples made from 3D-printed (PT3) and bulk (BT3) materials. The measured surface hardness values are shown in Figure 8.

The next step involved calculating the theoretical surface hardness distribution using the modified ECM method, assuming a given in-depth hardness function. Various realistic parameter combinations were applied in Equation (3) to describe the in-depth hardness distribution in the layer. For each case, the calculated surface hardness profile was compared to the measured values in Figure 8. The investigated parameter ranges were: *A*_1_ = 700–1400 HV, *x*_0_ = 5–15 μm, and *dx* = 1–10, and *A_2_* was kept constant at 535 HV according to measurement results given in Table 4. The whole calculation procedure is detailed in a previous study [44]. The best fit (R^2^ = 0.97) and the slightest deviation between the calculated and measured surface hardness values were obtained with the following parameter combination in Equation (3): *A*_1_ = 1125 HV, *A*_2_ = 535 HV, *x*_0_ = 8.4 μm, *dx* = 4.1. This parameter set is therefore assumed to represent the in-depth hardness distribution of the nitrided layer. By applying this parameter set, the red curve in Figure 7 illustrates the in-depth hardness (as a function of distance from the surface), and the blue curve in Figure 8 represents the surface hardness distributions (as a function of applied load) of the nitrided layer.

Based on the in-depth hardness distribution function, *H_d_*(*x*) determined in this way, the nitrided layer thickness belonging to the inflection point, and the nitrided hardness depth are *x_i_* = 8.4 μm and *x_NHD_* = 20.9 μm, respectively. According to Equation (3), the hardness values at these characteristic depths are 830 HV and 585 HV (535 + 50 HV), respectively. The hardness at the surface of the nitrided layer, i.e., at a depth of *x* = 0 µm, is 1025 HV.

The OM and SEM measurements carried out on the PT3 and BT3 nitrided target samples revealed an average thickness of the nitrided layer of *x_v_*~10 µm, falling within the range of 9–11 μm (Table 4).

It can be established that the values obtained by the different methods varied significantly. It is therefore essential to clearly indicate in each case the principle of interpreting the nitrided layer and to select the method of case depth determination carefully, based on specific requirements and consistent with particular application and economic considerations.

#### 3.3.4. Estimating the Composite Hardness of the Duplex-Treated, i.e., the Nitrided and PVD-Coated Samples

In the current research, the surface hardness of 3D-printed, simultaneously aged, and nitrided maraging steel samples (designated as P series) is compared to that of the reference Böhler W720 (1.6358), solution-annealed and heat-treated bulk specimens (designated as B series). However, as a result of the PVD coating, a significantly rougher surface quality was obtained compared to the nitrided state (see Table 4 and Figure 6c), which limits the applicability of surface hardness measurements.

Since the thickness of the PVD layer is only a few µm, it is advisable to use very low loads (e.g., 50–500 mN) during surface hardness testing. However, at this load level, the indentation depth in the high-hardness (2000–4000 HV) coating falls into the sub-µm range. Therefore, surface quality and surface homogeneity play a crucial role in ensuring the reliability of the measurements.

Results from preliminary hardness measurement series demonstrated that, for the examined samples, a minimum indentation depth of 4–5 μm—corresponding to an impression with a diagonal of at least 30 μm—is required to achieve reproducible surface hardness measurements. Considering the expected hardness values, it follows that a minimum load of 10 N (approximately 1 kgf) must be applied for reliable testing. Therefore, for the PVD-coated samples, the lower limit of the applied load range in the hardness testing series was set to 1 kgf. Composite hardness values corresponding to lower load levels were estimated using the modified ECM.

**Figure 9:**
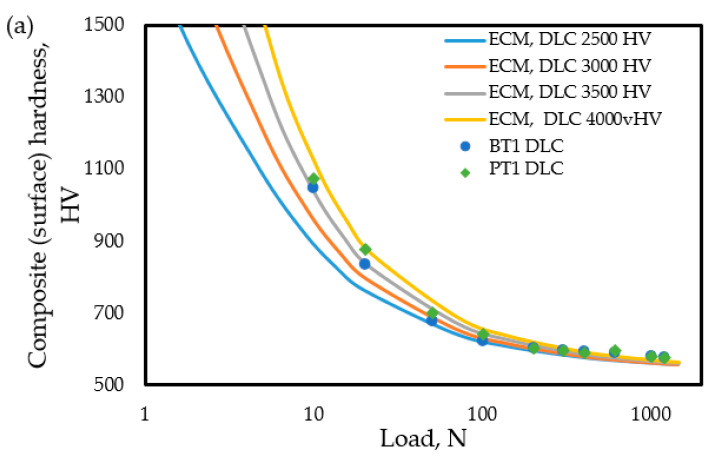

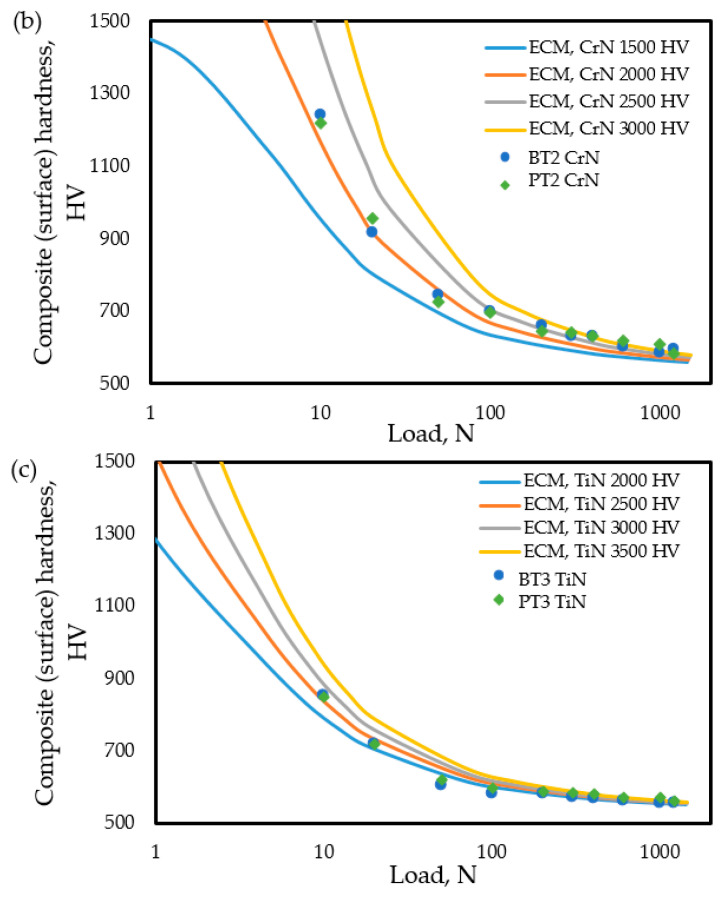
Composite hardness vs. loading force values obtained from measurements and the corresponding functions estimated by the ECM for duplex-treated surfaces coated with (**a**) 2.7 μm DLC, (**b**) 7.2 μm CrN, and (**c**) 2.1 μm TiN PVD layers.

In Figure 9 the individual measurement points represent the surface hardness measurement results as a function of the applied load determined on the duplex-coated specimens (the meaning and determination method of the continuous lines in Figure 9 is explained in the following paragraphs), with the following PVD coatings: a 2.7 μm thick DLC coating (samples PT1 and BT1, Figure 9a), a 7.2 μm thick CrN coating (samples PT2 and BT2, Figure 9b), and a 2.1 μm thick TiN coating (samples PT3 and BT3, Figure 9c). The as-built 3D-printed (P series) and bulk annealed (B series) samples exhibit very similar surface hardness values at all applied loads. In both series, the measured hardness tends to decrease as the load increases, indicating similar behavior during indentation testing.

In the following section, the method for calculating the theoretical composite surface hardness derived from an assumed in-depth hardness distribution is presented. The thickness of the PVD coatings is known based on measurements (Table 4), whereas the hardness of the coatings can vary within a wide range (Section 2.2). The in-depth hardness distribution of the nitrided layer beneath the PVD coating is also known, based on the procedure described in Section 3.3.3 (Figure 7). Accordingly, the parameters of the in-depth hardness distribution of the duplex layer (nitrided + PVD-coated), as illustrated in Figure 1a, can be considered known—except for the hardness values of the PVD coatings. The series of curves shown in the diagrams of Figure 9 represents the expected surface (composite) hardness functions calculated using the modified ECM, taking into account the bimodal layered structure and the combined effect of the nitrided layer and the PVD coating. Each curve in the series corresponds to a different PVD hardness value falling into the range characteristic of the particular PVD coating. The in-depth hardness distribution of the nitrided zone beneath the PVD layer was considered using the function presented in Figure 7.

Accordingly, in Figure 9a, for example, the bottom curve represents the calculated surface (composite) hardness as a function of the applied load estimated by the modified ECM for a sample with a 2.7 μm thick DLC coating possessing a hardness of 2500 HV, with an underlying nitrided layer as described by the function in Figure 7. Similar calculations were performed for DLC layers possessing hardness values of 3000, 3500, and 4000 HV. Based on the fit between the calculated curves and the measured data points, the hardness of the DLC layer is most likely to fall within the range of 3000–3500 HV. According to Figure 9b, the CrN layer appears to possess a hardness in the range of 2000–2200 HV, while Figure 9c suggests that the TiN layer likely has a hardness around 2500 HV.

## 4. Discussion

We have extensively investigated the simultaneous aging and surface treatment—high temperature, i.e., 530 °C cyanide-free salt bath nitriding and PVD coating, often used in industry—of parts produced by state-of-the-art 3D printing (SLM). Similarly treated, conventionally produced, bulk Böhler W720 (1.6358) steel parts were used as a reference. The comparison of the effect of the two different initial material conditions on the volume and surface treatment processes was carried out based on an analysis of the different hardness characteristics.

In general, no significant difference was observed in the hardness characteristics (surface hardness and in-depth subsurface hardness profile) between samples with a 3D-printed or bulk annealed initial condition throughout the complete surface treatment process. This observation is consistent with the results of preliminary experiments, indicating that, in terms of hardness and hardness evolution, the 3D-printed condition can be considered equivalent to the annealed state of the reference material within the thermal cycle of the surface treatment. Similar findings were reported by Frank et al. [54] for 1.2709 maraging steel.

Throughout the entire heat treatment cycle, the hardness of the base steel matrix in both investigated cases (P and B series) was 535 HV, which falls short of the targeted initial and expected hardness of around 590 HV. The primary reason for this is that the duration of preheating before nitriding in the industrial experiment was shorter (1 h) than planned (2 h) at 380 °C. As a result, the duration of the subsequent treatment step, i.e., 25-min holding time at a nitriding temperature of 530 °C, was insufficient to achieve the metallurgically attainable peak hardness at this temperature. This finding is also supported by the isothermal DSC measurements of Casati [55] on a maraging steel of similar composition. In their experiment—which did not include a preheating step—more than 60 min of holding at 540 °C was required to reach the maximum hardness.

There is some difference in the activation energies of the exothermic reactions during the precipitation hardening, considering the two initial conditions of the base materials, as indicated by the DSC results summarized in Table 3. The temperature intervals of the two exothermic processes, as well as the corresponding activation energy values, are in good agreement with the results of similar investigations [45,46,47,48,55]. However, surface hardness measurements performed on the nitrided (pre-PVD) condition of samples of PT3 and BT3 (Figure 8) confirm that this difference is negligible, at least in terms of hardness evolution.

An essential aspect of the findings is the consistency of the in-depth hardness distribution in the nitrided layer between the as-built and bulk reference samples. It suggests that the nitriding kinetics and nitrogen diffusion characteristics are not significantly affected by the initial microstructural differences stemming from additive manufacturing versus conventional processing. The similar thickness and gradient of the nitrided layers—confirmed by microscopy and ECM-based modeling—support this observation.

Accordingly, the nitrided states of samples PT3 and BT3 can be characterized by the same in-depth hardness distribution and case depth values (Figure 7). The in-depth hardness function of the nitrided layer is determined by surface hardness measurements and the application of modified ECM theory in this study. The shape of the in-depth hardness distribution function and the estimated surface hardness value are in good agreement with literature data [2,3,4].

The surface hardness measured after the PVD coating, although strongly dependent on the coating type and thickness, does not show any difference concerning the initial material condition (P or B). Based on Figure 9, it can be established that the surface (composite) hardness of the P and B samples with the same PVD coating depends on the applied load to a similar extent (Figure 9a–c). Consequently, the hardness values estimated by the modified ECM in the investigated load range can be described by the same function for coated materials systems with substrate materials of both initial conditions.

**Figure 10:**
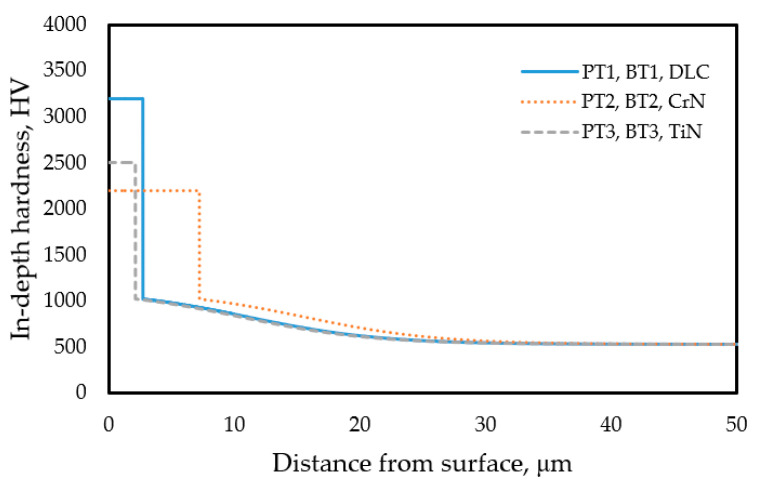
The estimated in-depth hardness distribution developed in the nitrided and PVD-coated target test samples, which had substrate materials of as-built, 3D-printed, and bulk, solution-treated maraging steel.

Based on the above results, the in-depth hardness distribution within the duplex layer can be estimated along a cross-section perpendicular to the surface, as illustrated in Figure 1a. The in-depth hardness distribution as a function of the distance from the surface for all three types of duplex coatings is shown in Figure 10.

These hardness distribution functions are consistent with the results of the surface hardness measurements. The near-surface plateau of each curve represents the hardness and thickness of the three PVD coatings that were applied. The hardness is assumed to be uniform throughout the entire thickness of a given PVD coating. On the duplex-treated pieces, the hardness distribution in the substrate material below the coating is identical to that determined on the nitrided pieces before PVD coating, as presented in Figure 7.

The connection between the in-depth hardness profiles presented in Figure 10 and the surface hardness functions shown in Figure 9 is described in the present study by the modified ECM.

## 5. Conclusions

This study aimed to develop and validate a simplified, industrially applicable heat treatment process for 3D-printed maraging steel components by combining precipitation aging and surface nitriding into a single step, and to characterize the resulting surface and subsurface hardness using experimental testing and analytical modeling. The first part of the paper describes the heat treatment technology applied for simultaneous precipitation aging and salt bath nitriding of the investigated 3D printed (SLM) maraging steel grade EOS MS1. Two sets of samples were heat-treated under industrial circumstances. The industrially applied heat treatment technology was essentially the same as we modeled it by preliminary DSC measurements, except for the duration of the preheating step. In the second part of the paper, the determination and evaluation of composite hardness values were discussed. The layer structure and surface hardness of the as-built 3D printed and bulk, solution-treated specimens were very similar. Based on the presented research, the following conclusions can be drawn:

- The temperature and duration of the heat treatment steps can be optimized for simultaneous aging and surface treatment.
- The optimal temperature for simultaneous aging is around 480 °C, while industrial salt bath nitriding requires a higher temperature, approximately 530 °C, at which the alloy would reach its maximum hardness in 20–25 min. However, this short holding time would only result in the formation of a very thin nitrided layer, approximately 10 µm thick.
- The preheating step of heat treatment applied under industrial circumstances also has to be taken into account because it can strongly affect the aging process.
- The samples after PVD coating can be characterized by a surface roughness of around Ra = 0.5 µm. The load applied during hardness testing must be adjusted to the surface roughness of the sample.
- The in-depth hardness distribution of a very thin nitrided layer can be characterised by a set of surface hardness measurements combined with the application of a modified ECM.
- The composite in-depth hardness distribution of a duplex-treated (nitrided + PVD-coated) layer can be characterised by a set of surface hardness measurements combined with the application of a modified ECM.
- The evaluation and interpretation of the test results are effectively supported by the mathematical modeling of the relationship between surface and in-depth hardness functions using, e.g., the applied modified ECM or other appropriate model.
- There is no significant difference between the surface composite hardness of 3D printed maraging steel (P series) and bulk annealed Böhler W720 (B series) samples.

The results demonstrate that the mechanical response—both in terms of peak hardness and in-depth hardness distribution—of 3D printed, as-built maraging steel is functionally equivalent to that of conventionally solution-treated bulk steel when processed via the proposed simultaneous heat treatment route.

One of the key methodological contributions of this work is the successful application of the modified expanding cavity model (ECM) to estimate in-depth hardness distributions from surface hardness measurements on ultra-thin nitrided and duplex layers.

The comparative hardness analysis of samples coated with DLC, CrN, and TiN PVD layers revealed that surface hardness is governed primarily by the coating material and thickness, rather than by the substrate condition (as-built or bulk).

Future work may focus on fatigue, wear, or tribological testing of duplex-coated parts with different base material conditions and explore the long-term stability of the PVD coatings under service conditions.

## Figures and Tables

**Table 1:** Chemical composition of the EOS MS1 powder and Böhler W720 bulk maraging steel.

Maraging Steel Grade	Shape of the Raw Material	Element, wt%							
		Ni	Co	Ti	Mo	Al	Mn	C	Si
EOS MS1	powder, grain size of *d* = 20–30 µm	18.1	9	0.7	4.8	0.1	≤0.1	≤0.03	≤0.1
Böhler W720	hot rolled commercial rod, *D* = 30 mm	18.5	9	0.7	5	0.1	≤0.1	≤0.03	≤0.1

**Table 2:** The hardness of 3D printed samples after different isothermal heat treatments.

3D Printed Sample		Treatment				Hardness, HV5 6 Measurements		
		Solution Annealing		Aging				
No	Condition	Temp., °C	Time, h	Temp., °C	Time, h	Average	St. dev.	Coeff. of var., %
PP1	as-built	–	–	–	–	340–375	13.7	3.8
PP2	as-built+aged	–	–	480	2.5	570	9.0	1.6
PP3	as-built+aged	–	–	480	4.5	601	11.3	1.9
PP4	annealed	830	1	–	–	280	6.0	2.1
PP5	annealed+aged	830	1	480	2.5	573	4.1	0.7
PP6	annealed+aged	830	1	480	4.5	609	4.4	0.7

**Table 3:** Temperature range and activation energy of the exothermic reactions identified during aging of the as-built and the annealed 3D-printed maraging steel.

Sample Condition	Temperature Range (°C) at a Heating Rate of 5 K/min		Activation Energy (kJ/mol)	
	Peak 1	Peak 2	Peak 1	Peak 2
As built	359.2–447.3	442.2–551.2	223.8	235.4
Solution annealed	356.7–440.6	438.1–549.3	324.2	258.1

**Table 4:** Surface layer characteristics of industrially heat-treated target samples after nitriding and PVD coating for the as-built 3D-printed (PT) and solution-annealed bulk (BT) initial condition groups.

Samples	Base Material	EOS MS1			Böhler W720		
	Initial Condition	3D Printed (No Annealing)			Bulk, Solution Annealed		
	Designation	PT1	PT2	PT3	BT1	BT2	BT3
Characteristics of the nitrided layer	Thickness by OM, µm	9	10	9	10	11	10
	Thickness by SEM, µm	9	10	10	9	10	10
	Surface roughness, *Ra*, µm	0.021	0.024	0.019	0.019	0.022	0.020
	*Rz*, µm	0.149	0.155	0.148	0.144	0.124	0.116
Characteristics of the PVD coating	Type of coating	DLC	CrN	TiN	DLC	CrN	TiN
	Thickness by OM, µm	2.1	7.1	1.6	1.9	7.4	1.8
	Thickness by SEM, µm	2.7	7.2	2.1	2.6	7.3	2
	Thickness by Calotest, µm	2.8	7.2	2.1	2.9	7.2	2.2
	PVD thickness average, µm	2.7	7.2	2.1	2.7	7.2	2.1
	Surface roughness, *Ra* µm	0.48	0.65	0.46	0.58	0.68	0.38
	*Rz*, µm	2.96	3.94	2.65	3.61	4.49	2.67
Substrate core hardness, HV5	Average	537	534	534	538	532	536
	St. Dev.	7.4	7.2	6.2	5.6	4.2	5.8
	Var. coeff., %	1.4	1.3	1.2	1.0	0.8	1.1

## Data Availability

The original contributions presented in this study are included in the article. Further inquiries can be directed to the corresponding author.

## Appendix A

**Table A1:** Experimental Matrix for the Applied Heat Treatments.

Test Phase	TEST	Sample ID	Sample Condition	Test Treatment	Test Objective	Observation	Conclusions/Decision
PRE-TEST	Bulk heat treatment	PP_1	as-built (3D printed)	no treatment	Comparing the effect of aging on the as-built and annealed 3D-printed steel	No significant difference in the bulk hardness of the annealed vs. as-built 3D printed samples after the same aging treatment. Also, the hardness of the as-printed sample is slightly higher than that of the annealed 3D-printed sample.	Utilize the 3D-printed samples in their as-built condition for the target heat treatment test to save time, energy, and costs.
		PP_2	as-built + aged	*T* = 480 °C; *t* = 2.5 h			
		PP_3	as-built + aged	*T* = 480 °C; *t* = 4.5 h			
		PP_4	solution annealed	*T* = 830 °C; *t* = 1 h			
		PP_5	solution annealed + aged	*T* = 830 °C; *t* = 1 h and *T* = 480 °C; *t* = 2.5 h			
		PP_6	solution annealed + aged	*T* = 830 °C; *t* = 1 h and *T* = 480 °C; *t* = 4.5 h			
	DSC test (I) with continuous heating up to T = 600 °C	PP_1.1	as-built (3D printed, untreated)	Continuous heating up to *T* = 600 °C with *v_h_* = 2, 5, and 10 K/min	Define the temperature range and activation energy of the precipitation-hardening	No significant difference in ΔT and E_a_ of the aging process for the as-built vs. solution annealed, 3D printed samples	Utilize the 3D-printed samples in their as-built condition for the target test to save time, energy, and costs.
		PP_4.1	solution annealed (T = 830 °C; t = 1 h) (3D printed)				
	DSC test (II) with interrupted heating.	PP_1.2	as-built (3D printed)	Heating up to *T* = 600 °C, with *v_h_* = 10 K/min, interrup-ting (fast cooling + reheating) at 450, 500, and 550 °C	Determine the relationship between the thermal history and the change in hardness.	HV5 microhardness of the samples for the DSC I and DSC II tests is similar. Interruption does not influence the kinetics of the aging process.	The interrupted DSC test is a suitable measuring technique for modeling the kinetics and analyzing the thermodynamics of the precipitation hardening process of 3D printed samples.
	DSC test (III) with interrupted heating	PP_1.3	as-built (3D printed)	Heating up to *T* = 600 °C, with *v_h_* = 10 K/min, interrupting (fast cooling + reheating) between 150 and 600 °C at every heat increment of 50 °C			
	DSC test (IV) with continuous heating.	PP_1.4	as-built (3D printed)	Continuous heating up to *T* = 500 °C, *v_h_* = 10 K/min	Comparative analysis of the hardness evolution with no interruption of heating. Defining the heat flow.	HV5 microhardness obtained after aging with continuous heating up to different temperatures is identical to that obtained from the interrupted DSC tests.	
		PP_1.5	as-built (3D printed)	Continuous heating up to *T* = 550 °C, *v_h_* = 10 K/min			
		PP_1.6	as-built (3D printed)	Continuous heating up to *T* = 600 °C, *v_h_* = 10 K/min			
DESIGNING THE TARGET-TEST	DSC test (V) at a constant temperature with interrupted holding	PP_1.7	as-built (3D printed)	Fast heating (*v_h_* = 150 K/s) followed by isothermal holding at *T* = 530 °C and *t* = 200 min, and interruption at *t* = 5, 10, 15, 30, 60, 90, 120, 180 min for measuring HV.	Defining the optimal duration of simultaneous nitriding and aging at the designed temperature.	The optimal precipitation-hardened structure, with maximum hardness, forms at a holding time of *t* = 15–25 min at a temperature of *T* = 530 °C. The expected nitrided layer thickness, however, is thin: 10–15 µm.	Use a holding time of *t* = 15–25 min for simultaneous nitriding and aging to achieve maximum hardness.
	DSC test (VI) modeling the target test with an interrupted preheating and nitriding cycle	PP_1.7	as-built (3D printed)	Steps (as planned for the target test): (1) Preheat: *T* = 380 °C; *t* = 2 h (2) Nitriding+aging: *T* = 530 °C; t = 25 min PVD coating: *T* = 300 °C; *t* = 8 h	Modeling the complete industrial heat treatment and analyzing the hardness evolution to design the optimal technological parameters for the target test.	The achievable max. hardness is 590 HV reached in 10 min from starting the nitriding step. Due to overaging during the PVD step, the final hardness is approximately 560 HV.	Realize target tests by applying the technological parameters used in the DSC (VI) design test.
TARGET-TEST	Complete industrial heat treatment to produce duplex-coated samples with a maraging steel substrate under different initial conditions.	PT_1	as-built (3D printed) DLC coated	(1) Preheat: T = 380 °C; *t* = 1 h (2) Nitriding+aging: *T* = 530 °C; t = 25 min PVD coating: T = 300 °C; *t* = 8 h for DLC, TiN, and CrN.	Comparing the surface layer architecture and hardness distribution in duplex-coated samples with maraging steel substrates of different initial conditions.	The hardness distribution in the surface layer of the coated composite material systems is not influenced by the type of the applied substrate materials (bulk or 3D printed) or their heat treatment (whether it is a conventional, multi-step treatment or a simultaneous nitriding + aging process)	The industrial heat treatment process for producing wear-resistant, duplex-coated maraging steel components is suggested to be modified by applying simultaneous nitriding and aging treatments, as well as using 3D-printed components in their as-built condition, rather than the current practice of using bulk, annealed initial base material.
		PT_2	as-built (3D printed) CrN coated				
		PT_3	as-built (3D printed) TiN coated				
		BT_1	bulk, solution annealed (*T* = 830 °C; *t* = 1 h) DLC coated				
		BT_2	bulk, solution annealed (*T* = 830 °C; t = 1 h) CrN coated				
		BT_3	bulk, solution annealed (*T* = 830 °C; t = 1 h) TiN coated				

Sample designation: PP—3D Printed for Preliminary test; PT– 3D Printed for the Target test; BT—Bulk sample for the Target test.
